# Educational video for self-care with arteriovenous fistula in renal patients: randomized clinical trial ^*^

**DOI:** 10.1590/1518-8345.6949.4185

**Published:** 2024-06-14

**Authors:** Natália Ramos Costa Pessoa, Jackeline Kérollen Duarte de Sales, Clemente Neves Sousa, Marcos Venícios de Oliveira Lopes, Cecília Maria Farias de Queiroz Frazão, Vânia Pinheiro Ramos

**Affiliations:** 1 Universidade Federal de Pernambuco, Recife, PE, Brazil.; 2 Scholarship holder at the Coordenação de Aperfeiçoamento de Pessoal de Nível Superior (CAPES), Brazil.; 3 Universidade do Porto, Centro de Investigação em Tecnologias e Serviços de Saúde, Rede de Investigação em Saúde (CINTESIS@RISE), Porto, Portugal.; 4 Escola Superior de Enfermagem do Porto, Porto, Portugal.; 5 Universidade Federal do Ceará, Fortaleza, CE, Brazil.

**Keywords:** Renal Dialysis, Arteriovenous Fistula, Self Care, Health Education, Nursing, Clinical Trial, Diálisis Renal, Fístula Arteriovenosa, Autocuidado, Educación en Salud, Enfermería, Ensayo Clínico, Diálise Renal, Fístula Arteriovenosa, Autocuidado, Educação em Saúde, Enfermagem, Ensaio Clínico

## Abstract

**Objective:**

to evaluate the effect of an educational video on the knowledge, attitude, and practice of self-care with arteriovenous fistula in patients undergoing hemodialysis treatment.

**Method:**

randomized controlled clinical trial, with two arms and single-blind. The intervention used an educational video on arteriovenous fistula self-care. The Fistula Self-Care Knowledge, Attitude, and Practice Scale was applied to 27 renal patients on hemodialysis in the control group and 28 in the intervention group at baseline, after seven and fourteen days. The data was analyzed using the Statistical Package for the Social Sciences software, using the chi-square test, Student’s t-test, Mann-Whitney test, and Friedman’s test with post-hoc analysis for multiple comparisons.

**Results:**

there were statistically significant differences in the knowledge and practice of self-care with the fistula at 0, 7 and, 14 days in the intervention (p= 0.004 and p<0.001, respectively) and control groups (p<0.001 for knowledge and practice). Attitude showed a significant difference at follow-up (p<0.001), but the post-hoc analysis did not confirm the significance obtained.

**Conclusion:**

patients’ knowledge and practice showed significant increases at follow-up in the control and intervention groups, while the increase in attitude was not significant in either group. Clinical trial, registration number: U1111-1241-6730.

## Abstract (es)

**Objetivo:**

evaluar el efecto de un video educacional en el conocimiento, actitud y práctica del autocuidado de la fístula arteriovenosa de pacientes en tratamiento de hemodiálisis.

**Método:**

ensayo clínico aleatorio controlado, con dos brazos y simple ciego. La intervención utilizó un video educacional sobre el autocuidado de la fístula arteriovenosa. Fue aplicada la Escala de conocimiento, actitud y práctica del autocuidado de la fístula en 27 pacientes renales en hemodiálisis del grupo control y en 28 del grupo intervención en la línea de base, después de siete y catorce días. Los datos fueron analizados con el *software* *Statistical Package for the Social Sciences* , con la aplicación de las pruebas Chi-cuadrado, T de Student, Mann-Whitney y Test de Friedman, con análisis *post-hoc* para comparaciones múltiples.

**Resultados:**

se verificaron diferencias estadísticamente significativas en el conocimiento y práctica del autocuidado de la fístula en el seguimiento de 0, 7 y 14 días de los grupos intervención (p= 0,004 y p<0,001, respectivamente) y control (p<0,001 para el conocimiento y práctica). La actitud presentó diferencia significativa en el seguimiento (p<0,001), pero el análisis *post-hoc* no confirmó la significación obtenida.

**Conclusión:**

el conocimiento y práctica de los pacientes presentaron aumentos significantes en el seguimiento en los grupos control e intervención, en cuanto que el aumento de la actitud no fue significativo en ninguno de los grupos. Ensayo clínico, número de registro: U1111-1241-6730.

## Abstract (pt)

**Objetivo:**

avaliar o efeito de um vídeo educacional no conhecimento, atitude e prática de autocuidado com a fístula arteriovenosa de pacientes em tratamento hemodialítico.

**Método:**

ensaio clínico randomizado controlado, com dois braços e unicego. A intervenção utilizou um vídeo educacional sobre o autocuidado com a fístula arteriovenosa. Foi aplicada a Escala de conhecimento, atitude e prática de autocuidado com a fístula em 27 pacientes renais em hemodiálise do grupo controle e 28 do grupo intervenção na linha de base, após sete e quatorze dias. Os dados foram analisados no *software* *Statistical Package for the Social Sciences* , com a aplicação dos testes qui-quadrado, T de Student, Mann-Whitney e Teste de Friedman com análise *post-hoc* para comparações múltiplas.

**Resultados:**

verificaram-se diferenças estatisticamente significantes no conhecimento e prática de autocuidado com a fístula no seguimento de 0, 7 e 14 dias dos grupos intervenção (p= 0,004 e p<0,001, respectivamente) e controle (p<0,001 para o conhecimento e prática). A atitude apresentou diferença significativa no seguimento (p<0,001), mas a análise *post-hoc* não confirmou a significância obtida.

**Conclusão:**

o conhecimento e prática dos pacientes apresentaram aumentos significativos no seguimento nos grupos controle e intervenção, enquanto o aumento da atitude não foi significativo em nenhum dos grupos. Ensaio clínico, número de registro: U1111-1241-6730.

## Introduction

 Chronic Kidney Disease (CKD) is one of the main causes of death from non-communicable diseases. Its prevalence has increased over time, so that it affects populations in different regions of the world unequally, probably as a result of differences in the demographic characteristics of the population, their comorbidities, and access to health resources ^(^ 1 ^)^ . In Brazil, around 139,691 individuals were undergoing kidney dialysis as a treatment for the disease in 2019, which represented an average increase of 6,881 patients (5.43%) compared to the previous year ^(^ 2 ^)^ . 

 Among the types of kidney dialysis, hemodialysis (HD) is the most widely used modality in Brazil (93.2%), requiring vascular access ^(^ 2 ^)^ . The Arteriovenous Fistula (AVF) is considered the most appropriate access when compared to grafts and catheters, as it has lower mortality rates and is related to a lower risk of infection ^(^ 3 ^)^ . 

 Despite being the most appropriate venous access for HD, the use of AVFs can be related to some complications, such as the presence of aneurysms, bleeding, ischemic neuropathies, lymphedema, and venous hypertensive events ^(^ 4 ^)^ . 

 In an attempt to reduce these complications, it is important for patients to develop self-care actions aimed at AVF ^(^ 5 ^)^ . Even so, the implementation of these actions has been lower than expected in many studies ^(^ 6 ^-^ 8 ^)^ . 

 In order to help the patient, the main guidelines available recommend carrying out educational actions aimed at them about vascular access ^(^ 9 ^-^ 12 ^)^ . In this context, educational technologies, as a potential strategy for professional practice, in addition to offering support, are able to provide the necessary support and guidance for care, as well as tending to increase the patient’s knowledge, with a view to promoting health and quality of life ^(^ 13 ^)^ . 

 Among educational technologies, video stands out as a strategy related to better health outcomes among diverse patient groups ^(^ 14 ^-^ 18 ^)^ . Furthermore, the use of video can benefit professionals, patients, and the management of hemodialysis clinics in terms of the health education process. This is because it allows the content to be replayed for the patient without the need for a health professional, which can optimize the use of these human resources in the face of the demand for existing activities ^(^ 15 ^)^ . 

 However, in order to verify the effect of isolated educational interventions or the combination of different interventions on the development of correct AVF self-care behaviors, it is important that they be evaluated through randomized clinical trials ^(^ 19 ^)^ . 

 In this sense, some studies have shown the positive effect of educational interventions among renal patients with an approach to AVF ^(^ 15 ^,^ 19 ^-^ 20 ^)^ and their effect on self-care practices with AVF ^(^ 15 ^,^ 19 ^)^ . 

 However, it is important to highlight the effect of these technologies not only on the practice of self-care but also on knowledge and attitude, since these concepts are interconnected. Knowledge refers to the apprehension of information with the potential to help maintain the functionality of the AVF; attitude represents the willingness to perform self-care, and is influenced by the patient’s convictions and feelings about access; and practice refers to actions developed by the patient, often based on their knowledge of the subject ^(^ 21 ^)^ . 

In view of the above, this study sought to answer the research question: Is the educational video “Care of the arteriovenous fistula” effective in improving the knowledge, attitude, and practice of self-care in patients undergoing hemodialysis due to an arteriovenous fistula?

To this end, the aim of this study was to evaluate the effect of an educational video on the knowledge, attitude, and practice of self-care with arteriovenous fistula of patients undergoing hemodialysis treatment.

## Method

### Study type

 This is a randomized, controlled, two-arm, single-blind clinical trial. The study followed the recommendations of the Consolidated Standards of Reporting Trials (CONSORT) for non-pharmacological interventions ^(^ 22 ^)^ and was registered in the Brazilian Clinical Trials Registry (ReBEC) database with the identification number U1111-1241-6730, which was approved in April 2020. 

### Study variables

 The variables collected were classified into two groups: the Dependent Variable group (equivalent to the outcome of the intervention), which comprises the scores of knowledge, attitude and practice of self-care with AVF among patients undergoing hemodialysis treatment, assessed by the results of the KAP (Knowledge, Attitude, and Practice) survey on the self-care of patients with AVF; and the Independent Variables group (explanatory and descriptive), which comprises A) Sociodemographic variables: age (in years); gender (female/male); marital status (with partner/without partner); schooling (in years of study); occupation (self-employed; employed/unemployed/retired/student); monthly *per capita* income (in *reais* ); health care plan (public health network/private health network); and, B) Clinical variables: complications with the current AVF (yes/no); presence of previous AVF (yes/no); length of hemodialysis treatment (in months); length of hemodialysis treatment with AVF (in months); length of hemodialysis treatment with current AVF (in months). 

### Intervention

 The intervention in this study was mediated by the use of the educational video “Care of the arteriovenous fistula”. This audiovisual resource was produced based on the precepts of Dorothea Orem’s General Nursing Theory and evaluated by experts represented by nurses and media professionals ^(^ 23 ^)^ . 

 The items evaluated by these experts were related to the concept of the idea, dramatic construction, rhythm, characters, dramatic potential, dialogues, visual style, target audience, and relevance. In addition to these, the media professionals also assessed the video’s functionality, usability, and efficiency. The items considered inappropriate were modified according to the experts’ suggestions ^(^ 23 ^)^ . 

 The educational technology is three minutes and seventeen seconds long and covers the self-care actions that patients should carry out before and after the arteriovenous fistula is made, during its use in hemodialysis, and in the prevention and monitoring of complications during access ^(^ 23 ^)^ . 

 The contents of the video regarding the pre-surgery of the AVF were the care taken to preserve the venous network of the arm chosen by the doctor. In the postoperative period, care is taken to dress the surgical wound and, during the use of the AVF for hemodialysis, the video illustrates actions that should be avoided on the arm, such as wearing watches, tight clothing, measuring blood pressure, sleeping on it, or carrying excess weight with it. For the prevention and monitoring of access complications, the precautions covered are routine checking of the AVF’s fremitus, washing the limb before HD, hemostasis at the end of therapy, and monitoring and treating complications such as hematomas, infections, steal syndrome, and thrombosis ^(^ 23 ^)^ . 

### Study location, period, and population

The study was carried out in the hemodialysis services of a northeastern capital. To allocate them to the control and intervention groups, four clinics were selected by drawing lots among all 13 establishments offering hemodialysis treatment. In this way, all the clinics had the same probability of being selected for the study by drawing lots without the intervention of the researchers. The study population was made up of patients undergoing hemodialysis at these services between July and November 2021.

### Selection criteria, sample, and sampling

 The sample calculation equation for two experimental means was used to determine the sample size ^(^ 24 ^)^ . A 95% confidence coefficient and 80% test power were defined. As for the means and standard deviation of knowledge, attitude, and practice of self-care with AVF, they were obtained from the application of a pilot study conducted in May 2021 with hemodialysis patients in the selected centers. 

 To define the pilot study sample, the same sample calculation equation was used for two experimental means ^(^ 24 ^)^ . However, we considered the average self-care behavior with AVF of 71% and a standard deviation of 13.6, as shown in the study by Sousa and collaborators (2017) ^(^ 25 ^)^ . It is important to note that self-care behavior is equivalent to the practice assessed in this study and there are no studies assessing the dimensions of knowledge and attitude with representative samples. 

 For the average number of self-care behaviors in the intervention group, the expectation of a 10% increase in the frequency of self-care was considered and the standard deviation was not altered by the Control Group (CG). To the sample value obtained, 77% was added, equivalent to possible losses during the continuation of the study. ET losses were estimated on the basis of a study ^(^ 26 ^)^ and took into account the rate of death, kidney transplantation, and change of dialysis modality or leaving the original clinic. 

Thus, the total sample defined was 52 patients allocated to the CG and 52 to the Intervention Group (IG), while the sample for the pilot test was 6 patients for each group (equivalent to 10% of the calculation obtained). It should be noted that 10 patients were recruited for each of the groups in order to take account of losses during data collection and 7 completed the entire follow-up. Based on the results of the pilot study, a new sample calculation was made.

As well as providing the necessary measurements for the sample calculation, the pilot study aimed to verify the need for adjustments to the data collection procedure. Patients undergoing hemodialysis at the clinics initially drawn for the control and intervention groups took part in this stage, with the aim of promoting conditions identical to the actual clinical trial in the pilot test.

The data collected for the pilot study was kept for the experimental study, as the necessary changes in the conduct of the research did not affect the quality of the collection content.

 The means and standard deviation found in the pilot study are shown in Table 1 . 

**Table 1:** - Mean and standard deviation scores for knowledge, attitude, and practice of self-care with AVF ^*^ in the control and intervention groups, at baseline and on the seventh day after intervention. Recife, PE, Brazil, 2023

**Follow-up** **Dimension**	**Base**		**Seventh day**	
	**CG** ^†^	**IG** ^‡^	**CG** ^†^	**IG** ^‡^
Knowledge	69±5.55	62.6±12.4	70±6.1	73.7±10.8
Attitude	19.5±1.22	18.8±2.8	19.1±1.6	18.3±2.2
Practice	24.3±5.6	23.5±3.5	25.8±5.2	24.3±4.2

* AVF = Arteriovenous Fistula;

† CG = Control Group;

‡ IG = Intervention Group

The sample calculation resulted in 47 patients for each of the groups, when considering knowledge, 10 patients for assessing attitude, and 12 for verifying practice.

 The inclusion criteria for the sample were: patients over the age of 18 using an AVF for hemodialysis treatment for at least six months, in order to allow patients to understand and carry out self-care actions with the AVF. Patients with some level of mental or cognitive disorder assessed by the Mini-Mental State Examination (MMSE) ^(^ 26 ^)^ and patients with a knowledge score measured in the pre-test higher than 76, which represents 80% of the maximum score for this dimension, were not included. Patients with a diagnosis of total hypoacusis, as described in their medical records or signaled by the attending physician, were also excluded from the sample because they were unable to understand the educational video. 

### Data collection instruments and procedures

 The first encounter with the patient took place during hemodialysis treatment, when cognitive status was assessed using the MMSE and then a socioeconomic and clinical questionnaire was administered to patients who met the inclusion and exclusion criteria. The evaluation of knowledge, attitude, and practice of self-care with AVF was carried out in the pre-test through telephone conversations and the application of a previously validated scale ^(^ 21 ^)^ . 

The Scale of Knowledge, Attitude, and Practice of Self-Care with AVF (ECAPA-FAV, from its acronym in Portuguese) has 31 items with scores ranging from 1 to 5, so the higher the score, the more adequate the patient’s knowledge, attitude, and practice. The knowledge items seek to identify how much the patient knows about self-care with the AVF, with questions such as “What do you know about signs that the fistula is infected?”. The attitude scale aims to identify the importance the patient attaches to this self-care through questions such as “How important do you think it is to know how to care for the fistula?”. Finally, questions such as “How often do you ET the habit of checking whether the arm of the fistula is red, hot, or has a secretion?” are contained in the practice scale with the intention of identifying the self-care activities carried out by patients.

 In its validation process, the scale was applied to renal patients dialyzing by AVF and subjected to exploratory factor analysis, which enabled its final structure to be obtained with explained variance and McDonald’s Omega values of 40.4%/0.896, 60.7%/0.843, and 36.9%/0.702 for knowledge, attitude, and practice, respectively ^(^ 21 ^)^ . 

Patients were randomized using conglomerates so that allocation to the control and intervention groups was defined by naturally occurring groupings. In the case of this study, the clusters were dialysis clinics. This type of randomization was chosen to avoid patients participating in the IG discussing the intervention with the CG members. Thus, the distance between the hemodialysis clinics allocated to the two groups was an obstacle to communication between the patients.

Patients were allocated to the control or intervention groups in two stages. Firstly, the hemodialysis clinics that would be part of each group were drawn by lot, so that the first two clinics drawn made up the intervention group and the next two were allocated to the control group. Once the clinics participating in the study had been determined, a list of patients dialyzing by AVF was requested to enable the selection of the participants for the study. The patients selected for the final sample were then drawn at random from each of the clinics drawn in the first stage. The draw was carried out by the main researcher, under the supervision of two members of the research group, in order to guarantee the suitability of the process.

After the pre-test, the intervention was carried out in IG. The video was played twice on the hemodialysis day following the pre-test, in order to optimize the assimilation of the information, since the patient may not be aware of all the instructions given on first contact. This took place during hemodialysis treatment, individually, using an electronic tablet with a 9.7-inch screen and a basic headset.

Both the control group and the Intervention group were subjected to the usual health education actions carried out in the hemodialysis units themselves, represented by information delivered verbally to the patients by the health professionals during the preoperative consultation for the AVF and during the hemodialysis session. The content of the information provided was similar to that contained in the educational video used as an intervention in this study, although there was no specific script to guide the delivery of this information.

The post-test was administered 7 and 14 days after the educational intervention in the case of patients allocated to the intervention group and 7 and 14 days after the pretest for patients in the control group. To this end, the same team responsible for the pretest contacted them by telephone and reapplied the ECAPA-FAV to the study participants. The face-to-face application of the scale in the pre-test lasted an average of 25 minutes, so the items on the scale were just read out to the participants, with no explanation of their content. If necessary, the person responsible for data collection was authorized to repeat the item as it was presented in the instrument.

For the post-tests, the ECAPA-FAV was applied by telephone, in which the researcher identified herself, mentioned the pre-test stage, and proceeded to read out the instructions for completing each item, repeating them if requested. For each item read, she waited for the participant’s response and moved on to the next item on the scale, ending the call by thanking them for their participation, making the average phone call last twenty minutes.

Patients who had been transplanted or who had died during the data collection period and patients who did not answer the phone to take the post-test after five attempts during the follow-up day and the three days afterward were considered to have dropped out or been lost.

It should be noted that the researcher responsible for playing the video was not blinded, since she applied the intervention and managed the data collection team. However, in order not to compromise the research results, the team responsible for administering the pre and post-test and the statistician responsible for analyzing the data were blinded.

### Data processing and analysis

The data was analyzed using the Statistical Package for the Social Sciences for Windows (SPSS) software, version 20.0. Initially, the relative and absolute frequencies of the qualitative variables and the statistical measures of minimum, maximum, mean, and standard deviation of the quantitative variables were calculated.

The homogeneity between the representatives of the control and intervention groups at baseline and the assessment of differences between the knowledge, attitude, and practice of self-care between the groups were checked using the chi-square test for the qualitative variables and the Student’s t-test and Mann-Whitney test. The variables were quantitative. To check for normality, the Kolmogorov-Smirnov test was applied to the entire sample of patients (members of the control and intervention groups).

The Friedman test was used to compare the scores for knowledge, attitude, and practice of self-care with AVF at baseline (D0), on the seventh (D7) and fourteenth (D14) days. In cases where the Friedman test showed statistical significance, a post-hoc analysis was carried out for multiple comparisons. The Shapiro-Wilk test was used to assess the normality of the scores in each of the groups. A 5% significance level was used for all conclusions.

### Ethical aspects

This study was approved by the Research Ethics Committee (CEP) of the Federal University of Pernambuco, under opinion number 3.555.992. Data collection began only after approval from the REC and the signing of the Free and Informed Consent Term (FICT) by the study participants.

It should also be noted that, as the study used headphones connected to the tablet to apply the intervention, certain precautions were taken to reduce discomfort caused by noise and the risk of infection caused by sharing the device. An intermediate volume was used for the sound during video playback, which could be changed according to the participant’s wishes. As for the risk of cross-infection, this was reduced by antisepsis with 70% alcohol before and after each use of the headphones.

## Results

**Figure 1:**
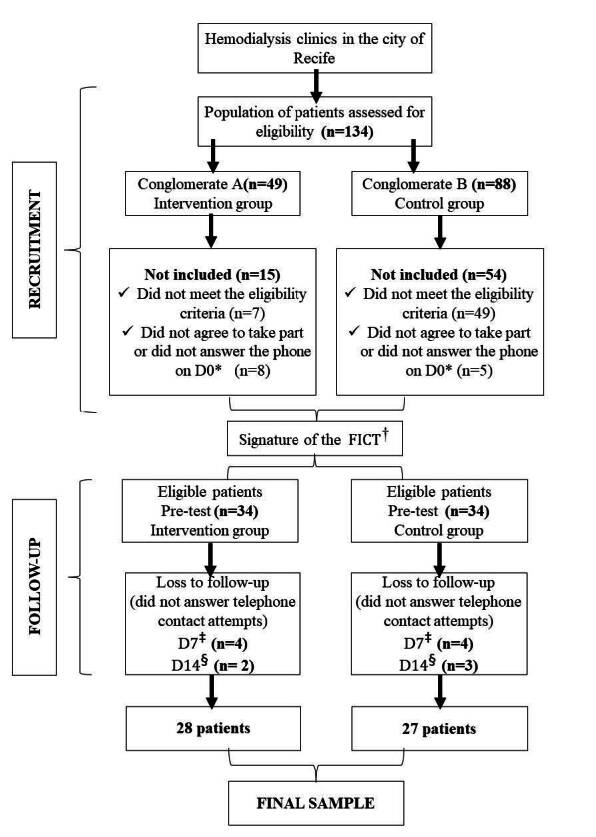
- Flow diagram of the stages of the experimental study to assess the knowledge, attitude, and practice of self-care with the AVF ^‖^ of patients undergoing hemodialysis treatment. Recife, PE, Brazil, 2023

 The final sample was 28 patients in the intervention group and 27 patients in the control group, which was lower than planned for the knowledge outcome (47/47) and higher for the attitude (10/10) and practice (12/12) outcomes. Details of the recruitment and follow-up of participants in the control and intervention groups are shown in Figure 1 . 

 The control and intervention groups were homogeneous in terms of gender, marital status, health insurance, occupation, presence of complications, current AVF, age, schooling, *per capita* income, and length of HD treatment with AVF and current AVF ( Table 2 ). 

*D0 = Day zero; ^†^ FICT = Free and Informed Consent Term; ^‡^ D7 = Day seven; ^§^ D14 = Day fourteen; ^‖^ AVF= Arteriovenous Fistula

**Table 2:** - Sociodemographic and clinical characteristics of participants. Recife, PE, Brazil, 2023

**Variables**	**Groups**			**p-value**
	**TOTAL** **(n=55)**	**Intervention** **(n=28)**	**Control** **(n=27)**	
	**n (%)**	**n (%)**	**n (%)**	
**Sex**				
Male	37(67.3)	19(67.9)	18(66.7)	1.000 ^*^
Female	18(32.7)	9(32.1)	9(33.3)	
**Marital status**				
With partner	33(60)	16(57.1)	17(63)	0.785 ^*^
Without partner	22(40)	12(42.9)	10(37)	
**Health plan**				
Public	23(41.8)	14(50)	9(33.3)	0.277 ^*^
Private health plan	32(58.2)	14(50)	18(66.7)	
**Occupation**				
Retired/Beneficiary	32(58.2)	18(64.3)	14(51.9)	0.418 ^*^
Other	23(41.8)	10(35.7)	13(48.1)	
**Complications with** **current AVF** ^†^				
Yes	25(45.5)	11(39.3)	14(51.9)	0.422 ^*^
No	30(54.5)	17(60.7)	13(48.1)	
**Presence of previous AVF** ^†^				
Yes	18(32.7)	8(28.6)	10(37)	0.573 ^*^
No	37(67.3)	20(71.4)	17(63)	
	**Md** ^‡^ **(p25-p75)** ^th^	**Md** ^‡^ **(p25-p75)** ^§^	**Md** ^‡^ **(p25-p75)** ^§^	**p-value**
**Age**	54 (44-66)	52(42-65)	54(46.25-67)	0.712 ^ǁ^
**Education**	12 (10-16)	14(12-17)	12(8-14.75)	0.052 ^¶^
**Monthly *per capita* income** ^**^	1650 (916-3300)	2750(687.5-4400)	1375(1100-2681)	0.307 ^¶^
**Time of HD** ^††^	36 (22-74)	48(28-84)	33.5(19.75-64.5)	0.619 ^¶^
**Time of HD** ^††^ **by AVF** ^†^	33 (12-63)	36(11-63)	26.5(12-64.5)	0.919 ^¶^
**Time of current AVF** ^†^	24 (10-54)	24(8-54)	24.5(11-57.5)	0.781 ^¶^

* Chi-square test;

† AVF = Arteriovenous Fistula;

‡ Md = Median;

th p25-p75 = 25 percentile-75 percentile;

ǁ Student’s t-test;

¶ Man-Whitney U-test;

** Monthly = In months;

†† HD = Hemodialysis

 There was also homogeneity between the knowledge, attitude, and practice scores at baseline (D0). Regarding the difference between the groups in knowledge, attitude, and practice of self-care with AVF on the seventh (D7) and fourteenth day (D14), statistical significance was only found between the practice scores on the seventh day of follow-up, as shown in Table 3 . 

 When comparing the scores for knowledge, attitude, and practice of self-care with AVF within each group during follow-up, there was statistical significance between the knowledge and practice of the intervention and control groups. Attitude was only significant in the intervention group, as shown in Table 4 . 

**Table 3:** - Intergroup comparison of knowledge, attitude, and practice of self-care with AVF ^*^ of participants at baseline, after 7 and 14 days of intervention. Recife, PE, Brazil, 2023

**Variables**	**Groups**		**p-value**
	**Intervention**	**Control**	
	**Median ranks**		
**Knowledge**			
Baseline (D0) ^†^	26.95	29.09	0.656 ^‡^
7 days (D7) ^§^	29.13	26.83	0.656 ^‡^
14 days (D14) ^‖^	30.63	25.28	0.260 ^‡^
**Attitude**			
Baseline (D0) ^†^	28.48	27.50	0.807 ^¶^
7 days (D7) ^§^	27.89	28.11	0.958 ^¶^
14 days (D14) ^‖^	30.48	25.43	0.200 ^¶^
**Practice**			
Baseline (D0) ^†^	29.21	26.74	0.659 ^‡^
7 days (D7) ^§^	23.71	32.44	**0.043** ^¶^
14 days (D14) ^‖^	30.04	25.89	0.335 ^¶^

* AVF = Arteriovenous Fistula;

† D0 = Baseline;

‡ Student’s t-test;

§ D7 = After 7 days;

‖ D14 = After 14 days;

¶ Mann-Whitney U-test

**Table 4:** - Intra-group comparison of knowledge, attitude, and practice of self-care with AVF ^*^ of participants at baseline (D0 ^†^ ), after 7 and 14 days of intervention. Recife, PE, Brazil, 2023

**Variables**	**Groups**					**Percentage 75%**
	**Intervention**			**Control**		
	**MD** ^‡^	**Percentage 25%**	**Percentage 75%**	**MD** ^‡^	**Percentage 25%**	
**Knowledge**						
Baseline (D0 ^†^ )	68	56.25	77.5	55	48	70
7 day (D7 ^§^ )	76	59	86.25	66	54	76
14 day (D14 ^‖^ )	76	59	86.25	70	54	83
**p-value** ^¶^	**0.004**			**<0.001**		
**Attitude**						
Baseline (D0 ^†^ )	19	16.25	20	17	19	20
7 day (D7 ^§^ )	20	19	20	17	19	20
14 day (D14 ^‖^ )	20	19	20	17	19	20
**p-value** ^¶^	**<0.001**			0.630		
**Practice**						
Baseline (D0 ^†^ )	20.5	18	23	22	19	27
7 day (D7 ^§^ )	20.5	18	23	23	20	27
14 day (D14 ^‖^ )	35	32	38.75	35	31	38
**p-value** ^¶^	**<0.001**			**<0.001**		

* AVF = Arteriovenous Fistula;

† D0 = Day zero;

‡ MD = Median;

§ D7 = Day seven;

‖ D14 = Day fourteen;

¶ p-value = Friedman test

Post-hoc analysis for multiple comparisons showed significant changes between patients’ knowledge at baseline and day 7 in the control group (p=0.001) and intervention group (0.048) and at baseline and day 14 for both groups (p= 0.001 in the control group and p=0.048 in the intervention group). No differences were observed between the seventh and fourteenth day in the two groups.

Regarding the attitude toward self-care among patients in the intervention group, the post-hoc test for multiple comparisons did not confirm statistical significance after applying the video.

On the other hand, the practice of self-care with the AVF showed a statistically significant difference between the scores measured at baseline and on the fourteenth day in the control (p<0.001) and intervention (p<0.001) groups. Differences were also found between practice on the seventh and fourteenth day in both groups (p=0.001 in the control group and p<0.001 in the intervention group). There was no significant difference between self-care practice at baseline and the seventh day of follow-up in the two groups analyzed.

## Discussion

 Self-care is defined by Dorothea Orem in her theory as the actions taken by individuals for their own benefit in order to maintain life, health, and well-being. When these actions are carried out properly, they can help maintain the structural integrity and functioning of the human body ^(^ 28 ^)^ . 

 Facilitating factors or barriers can be found in the development of these actions by patients. ET should be explored by nurses in order to promote motivation and increase patients’ health literacy about the symptoms of chronic kidney disease, favoring independent self-management ^(^ 29 ^)^ . 

 It should be noted that health education actions aimed at patients about vascular access are recommended by the main guidelines available ^(^ 9 ^-^ 12 ^)^ . In addition, the adoption of these actions, when they take a patient-centered approach, with an analysis of their knowledge needs, can make them the protagonists in clinical decision-making regarding their state of health ^(^ 19 ^)^ . 

 The evaluation of the effect of an educational intervention for self-care with AVF has been shown in two high-impact studies and in only one of them was the intervention of an educational video ^(^ 15 ^,^ 19 ^)^ . 

 The first study evaluated the effect of a structured action based on a multi-method approach with theoretical and practical stages and the use of writing, listening, and visual stimulation. The patients evaluated showed an improvement in self-care behaviors with AVF, both in terms of managing signs and symptoms and preventing complications with the fistula ^(^ 19 ^)^ . 

 The use of health education based on an educational video was found to generate a significant increase in scores for self-care behaviors with AVF after two and four weeks. However, a significant increase in self-care behaviors was also found in the control group, which received a face-to-face intervention with a verbal explanation of access care ^(^ 15 ^)^ . 

A similar result was found in this study since the measure of self-care practice showed differences at baseline on the fourteenth day and on the seventh and fourteenth days after in the control and intervention groups. However, no differences were observed between the baseline and the first seven days after playing the video.

With regard to knowledge, there was a statistical difference between patients in the intervention group at baseline and on the seventh and fourteenth days of follow-up, but there was no difference between knowledge measured on the seventh and fourteenth days.

 This result may be related to the fact that educational videos, when used in hospital environments, are more effective in improving short-term health literacy than in changing the patient’s behavior and lifestyle ^(^ 30 ^)^ . On the other hand, it is considered that hemodialysis treatment allows a bond with the health team because it is a continuous outpatient therapy, so the educational video can be played repeatedly, especially in the idle moments of the therapy itself. 

 With regard to changes in the practice of self-care, the mechanisms that are effective in modifying health behavior are still little known. This phenomenon needs to be better understood in order to clarify the relationship between health behaviors and the factors that motivate them to take place ^(^ 31 ^)^ . 

 To do this, it is necessary to measure such behaviors and analyze their predictors and theoretical explanations of co-occurrences. Psychological, socio-cognitive, environmental, and political variables and mechanisms can influence changes in health behaviors and should be analyzed in correlational and interventionist studies ^(^ 31 ^)^ . 

The influence of these mechanisms may require greater adaptation from the patient, so it may take longer after the intervention has been applied for changes in self-care practices to be observed. This may justify the statistically significant difference between the practice measured at baseline and fourteen days after the educational video was applied.

 With regard to patient attitude, although no significant differences were confirmed between the scores measured before and after the educational video was used, it is important to note that the patients already had high levels of attitude toward self-care with the AVF even before the intervention was used. The fact that patients see the fistula as essential for the continuity and success of the treatment, as well as for their survival ^(^ 32 ^)^ , may have contributed to the high attitude scores found. 

 It is noteworthy that educational initiatives must involve the provision of information about the functioning, preservation and self-care behaviors of the AVF. It is necessary to consider the needs and doubts of patients and their families in order to promote improved communication and encouragement in maintaining self-care activities ^(^ 32 ^)^ . 

 Therefore, in addition to an educational component, training needs to include support interventions that promote the development of coping skills to deal with the demands of AVF and its negative impacts on the patient’s life ^(^ 32 ^)^ . 

This understanding can help professionals conduct educational actions that promote critical thinking in patients, favoring the acquisition of appropriate self-care practices based on a positive attitude towards AVF.

In addition to the positive results among patients in the intervention group, a significant increase in knowledge was also observed among patients in the control group, measured at baseline and after seven and fourteen days of follow-up. This may have occurred because measuring knowledge by reading the items on the ECAPA-FAV at baseline may have encouraged patients to seek appropriate information on self-care with vascular access.

 Ensuring that adults with chronic conditions have access to information can increase their confidence in carrying out self-care activities. The patient’s search for this information tends to help increase their confidence in reporting their concerns to the doctor and in understanding when health care should be sought from professionals in the area ^(^ 33 ^)^ . 

In view of the above, this study may have contributed to the acquisition of information on self-care with AVF, both among the patients in the intervention group and those in the control group. It provided clarification for the participants in the first group through the educational video and may have encouraged the participants in the second group to seek knowledge on the subject.

Furthermore, despite the positive effect of the isolated application of the video on the knowledge and practice of self-care with AVF among patients undergoing hemodialysis treatment, it is essential that it is also applied as part of more complex educational actions. These actions should consider motivational approaches, promoting active listening to the patient in order to identify and resolve existing doubts about self-care with vascular access, as well as identifying the factors that help and hinder its implementation.

It can be seen that the losses during the study’s follow-up resulted in a final sample below that was planned for the knowledge outcome, equivalent to a test power of 56%, which limited the interpretation of the results to knowledge of self-care with AVF. In addition, the use of self-assessment to measure knowledge about self-care with AVF may have had an impact on the results achieved, since people who know little about a subject tend to overestimate their own knowledge, while those who are considered experts tend to underestimate it (cognitive bias).

Using the video individually or as part of more complex interventions can help stimulate the patient’s interest in the subject, as well as prompt a broader discussion of the subject. In addition, the educational video can be used continuously during hemodialysis treatment, which is considered idle time for patients. It can be played in the hemodialysis room in order to reach a greater number of people at the same time since many services have TV sets that can play the proposed educational technology.

It is suggested that new clinical studies be conducted with the application of other educational interventions, providing a longer follow-up period to assess their effect on knowledge, attitude, and self-care practice over longer periods. It is also pertinent to verify the effect of the technology on the functionality of the vascular access by evaluating the parameters of the adequacy of the AVF, such as satisfactory blood flow, venous pressure, and urea clearance, as well as evidence of complications such as stenosis, thrombosis, and infections.

## Conclusion

Significant increases in the knowledge and practice of renal patients were identified during follow-up in the control and intervention groups. With regard to attitude towards self-care, no statistical significance was observed in either group. It is believed that the changes in knowledge and practice among participants in the control group may have been motivated by the fact that reading the items on the measurement scale used in this study encouraged patients to look for appropriate information on the subject.

This study’s results may be useful for the care of renal patients on hemodialysis and the management of services, since it makes the educational video available as an evaluated product, which may contribute to the acquisition of self-care behaviors with AVF. In addition, the video as educational technology offers the advantage of allowing the content to be reproduced repeatedly in the dialysis room itself and without the need for a health professional to show it.

In the field of research and teaching, this study contributes to the development of scientific knowledge on the promotion of self-care in chronic renal patients, as well as providing scientific evidence on the effectiveness of an educational strategy to promote self-care for chronic renal patients. Its results can be used to support further research on the subject, to improve the quality of life and adherence to treatment for patients with chronic renal failure, and to guide teaching practice.
